# The presence of primary sclerosing cholangitis in patients with ileal pouch anal- anastomosis is associated with an additional risk for vitamin D deficiency

**DOI:** 10.1093/gastro/gov035

**Published:** 2015-08-19

**Authors:** Andre Fialho, Andrea Fialho, Gursimran Kochhar, Bo Shen

**Affiliations:** ^1^Department of Internal Medicine, Cleveland Clinic Foundation, Cleveland, OH, USA and; ^2^Department of Gastroenterology and Hepatology, Cleveland Clinic Foundation, Cleveland, OH, USA

**Keywords:** inflammatory bowel disease, primary sclerosing cholangitis, ileal pouch-anal anastomosis, vitamin D deficiency

## Abstract

**Objective:** Vitamin D deficiency is common in patients with ileal pouch-anal anastomosis (IPAA) for ulcerative colitis (UC). Whether vitamin D levels are further lowered in patients with concomitant IPAA and primary sclerosing cholangitis (PSC) is not known. The aim of this study was to evaluate the presence of PSC as a risk factor for vitamin D deficiency in patients with UC and IPAA.

**Methods:** In this case control study, 74 patients with concurrent IPAA and PSC were included in the study group, and 79 patients with IPAA, but without PSC, served as controls. Forty-four variables were analyzed. Univariate analysis and multivariate analysis with stepwise logistic regression were performed.

**Results:** A total 153 eligible patients were included, with 74 (48.4%) in the study group and 79 (51.6%) in the control group. Vitamin D level in the study group was 18.9 ± 1.4 ng/dL compared with 30.3 ± 1.7 ng/d in the control group (*P* = 0.011). Vitamin D deficiency (≤ 20 ng/dL) was present in 65 (42.5%) patients. PSC occurred in 49 (75.4%) of the 65 patients with vitamin D deficiency. In the multivariate analysis, only the presence of PSC (odds ratio [OR]: 7.56; 95% confidence interval [CI]: 2.39–24.08; *P = *0.001) and vitamin D supplementation (OR: 2.58; 95% CI: 1.57–9.19; *P = *0.018) remained associated with vitamin D deficiency.

**Conclusion:** The presence of PSC was found to be an independent risk factor for vitamin D deficiency in UC patients with IPAA. These patients should be routinely screened and closely monitored for vitamin D deficiency.

## Introduction

Total proctocolectomy with ileal pouch-anal anastomosis (IPAA) is a common surgical option for the treatment of ulcerative colitis (UC) that it is unresponsive to medical treatment or other conditions such as familial adenomatous polyposis and cancer. Metabolic disorders have been extensively reported in IPAA including anemia, iron deficiency, vitamin B_12_ deficiency, vitamin D deficiency, bone loss and nephrolithiasis [1, 2]

Primary sclerosing cholangitis (PSC) is the most common liver disorder associated with inflammatory bowel disease (IBD). It is more common in UC than in Crohn's disease (CD), with prevalence of 8% and 1%–3%, respectively [3, 4, 5]. The frequency of vitamin D deficiency in patients with PSC with and without end-stage liver disease has been shown to be 14% and 57%, respectively [6]. Both cholestatic liver disease and end-stage liver disease have been associated with vitamin D deficiency. Low vitamin D levels have been shown to be present in chronic liver disease with a prevalence range of 64%–92% [7–9]. The mechanism responsible for vitamin D deficiency in liver disease is still unknown, but it is thought to be multifactorial and possibly associated with decreased intestinal absorption, decreased outdoor activities in the setting of increased fatigue and nutritional deficiency [10, 11].

Vitamin D deficiency is also commonly seen in patients with IBD [12–15], with the prevalence of vitamin D deficiency reported to be as high as 70% in patients with CD, [16] and up to 45% in patients with UC. [15]. Vitamin D deficiency has also been shown to be common in IBD patients submitted to surgery such as those with UC and IPAA. In these patients, below-normal vitamin D levels occur in 67% of cases, while vitamin D deficiency occurs in approximately 20% of the cases.

Vitamin D levels have not been studied in UC patients with IPAA and concomitant PSC. The presence of PSC, a cholestatic liver disorder, could potentially further burden vitamin D levels in patients with UC and IPAA, who have already been shown to have high rates of vitamin D deficiency. We hypothesized that patients with IPAA and PSC would a dual risk for vitamin D deficiency. The aim of this study was to evaluate if the presence of PSC is a risk factor for vitamin D deficiency in patients with UC and IPAA.

## Patients and Methods

### Patients

The medical records of 1221 patients from the Pouchitis Registry at the Cleveland Clinic were reviewed. Of these, 153 eligible patients were identified, of whom 74 had PSC. Data were collected retrospectively from the years 2001–2014. This case control study was approved by the Cleveland Clinic Institutional Review Board.

### Inclusion and exclusion criteria

Inclusion criteria were patients with (i) UC and with or without PSC and (ii) serum 25(OH)D_3_ measurement after colectomy.

Exclusion criteria were (i) IPAA for familial adenomatous polyposis or other non-IBD conditions and (ii) patients with PSC who had been submitted to liver transplantation.

### Study and control groups

After reviewing the medical records of 1221 patients from our Pouchitis Registry, 74 patients with PSC fulfilled the inclusion criteria (study group). From the remaining 1147 patients, 79 eligible age-matched controls (control group) were identified and included in the study.

### Demographic and clinical variables

Twenty eight variables were analyzed. Variables known to impact vitamin D status were assessed. Demographic variables included age, sex, race, body mass index, smoking status and geographical location (north *vs* south). Definition of geographical location was as follows: a northern location was considered above the latitude of 37 degrees north, and a southern location was considered below the latitude of 37 degrees north. The presence or absence of PSC diagnosis was also evaluated.

Laboratory variables consisted of blood levels of 25(OH)D_3_, hemoglobin, iron, ferritin, transferrin, albumin, aspartate aminotransferase, alanine aminotransferase, bilirubin, alkaline phosphatase, C-reactive protein, creatinine, calcium, phosphorus and parathyroid hormone.

Medication use was documented, including antibiotics, corticosteroids (including oral budesonide), and non-steroidal anti-inflammatory drugs (NSAID). Supplementation for calcium and vitamin D was also evaluated.

Blood levels of 25(OH)D_3_ were used to assess vitamin D status. Levels of 25(OH)D_3_ ≥ 30 ng/ml were considered normal. Low levels of vitamin D were considered when 25(OH)D_3_ was < 30 ng/ml. Low vitamin D levels were further subdivided into vitamin D insufficiency when 25(OH)D_3_ levels were between 21 and 29 ng/dl and vitamin D deficiency when 25(OH)D_3_ levels were ≤ 20 ng/dl.

### Outcome measurements

The primary outcome was to determine the frequency of vitamin D deficiency in PSC patients with UC and to evaluate the association of PSC as an independent risk factor for vitamin D deficiency in UC patients with IPAA.

### Statistical analysis

Statistical analysis was conducted using the SPSS software version 22 (IBM SPSS Statistics for Windows, Version 22.0. Armonk, NY: IBM Corp). Mean ± SD, or N (%) was used to present continuous variables. Continuous variables were compared using the Student *t* test (or the Wilcoxon rank sum test, as appropriate). Chi-square test (or the Fisher exact test, as appropriate) was used to compare categorical variables. Univariate analysis was used to identify potential risk factors for vitamin D deficiency.

Factors identified as being significant (*P* < 0.05) in the univariate analysis, along with known risk factors for vitamin D deficiency, were included in the multivariate logistic regression analysis. Stepwise approach was used for sifting through large numbers of potential independent variables.

## Results

This study included 153 patients. Of these, 74 (48.4%) had UC, IPAA and PSC (study group), and 79 (51.6%) had UC and IPAA only. The mean age of the overall population studied was 51.4 ± 1.2 years. The mean age of patients with and without PSC was 48.8 ± 13.8 years and 52.8 ± 16.0 years, respectively (*P* *=* 0.150). There were 69 females (45.1%) in the overall population, with 27 (36.5%) in the PSC subgroup and 42 (53.2%) in the subgroup without PSC (*P* *=* 0.051).The mean vitamin D level in the overall population studied was 24.8 ± 1.5 ng/dL. The mean vitamin D level in patients with PSC compared with patients without PSC was 18.9 ± 1.4 ng/dL and 30.3 ± 1.7 ng/dL, respectively (*P* *=* 0.011). Vitamin D deficiency (levels ≤ 20 ng/dL) was present in 65 (42.5%) of the 153 patients, while 75.4% (49/65) of the patients with vitamin D deficiency had PSC.

### PSC patients *vs* non-PSC patients

When comparing the subgroup of patients with PSC to the patients without PSC, the prevalence of low levels of vitamin D was 91.9% (68/74) *vs* 73.4% (58/79), respectively (*P* < 0.003). The prevalence of vitamin D deficiency was 66.2% (49/74) *vs* 20.3% (16/79) *P* < 0.001 in patients with PSC and without PSC, respectively.

In addition, higher levels of aspartate aminotransferase (75.7 ± 10.9 *vs* 23.8 ± 1.0 U/L, *P* < 0.001), alanine aminotransferase (75.9 ± 9.2 *vs* 21.4 ± 1.3 U/L, *P* < 0.001), bilirubin (2.4 ± 0.5 *vs*. 0.5 ± 0.1 mg/dL, *P* < 0.001) and alkaline phosphatase (229.8 ± 25.3 *vs* 76.2 ± 3.1 U/L, *P* < 0.001) were found in PSC patients, as expected. The albumin levels were not different between the groups (3.8 ± 0.1 *vs* 4.5 ± 0.3 mg/dL, *P* = 0.530).

### Risk factors for vitamin D deficiency

This study was further subdivided in two separate samples based on the presence or absence of vitamin D deficiency (25(OH)D_3_ ≤ 20 ng/dl), and statistical analyses were performed to evaluate possible risk factors associated with vitamin D deficiency.

In the univariate analysis, the following variables were associated with vitamin D deficiency: higher levels of total bilirubin, aspartate aminotransferase, alanine aminotransferase, alkaline phosphatase and creatinine; lower levels of calcium and transferrin; use of vitamin D supplementation, NSAID and corticosteroids and the presence of PSC as shown in **Table 1****.**

**Table 1.** Demographic, clinical and laboratory characteristics of patients with and without vitamin D deficiency

Variables	All cases (N = 153)	Vitamin D ≤20 ng/dL (N = 65)	Vitamin D >20 ng/dL (N = 88)	*P*
*Mean age, years*	51.40 ± 1.22	53.06 ± 1.76	50.17 ± 1.66	0.257
*BMI, kg/m2*	25.66 ± 0.64	25.70 ± 0.75	25.60 ± 1.03	0.382
*Race, n (%)*				
* Non-Hispanic*	2	1 (1.5%)	1 (1.1%)	0.829
* Hispanic*	151	64 (98.5%)	87 (98.9%)	
*Season at vitamin D measure, n (%)*				
* Winter*	51	29 (44.6%)	22 (25.0%)	0.090
* Autumn*	32	11 (16.9%)	21 (23.9%)	
* Spring*	36	13 (20.0%)	23 (26.1%)	
* Summer*	34	12 (18.5%)	22 (25.0%)	
*Smoking, n (%)*				
* Never*	119	52 (80.0%)	67 (76.1%)	0.837
* Ex*	31	12 (18.5%)	19 (21.6%)	
* Smoker*	3	1 (1.5%)	2 (2.3%)	
*Northern location, n (%)*	134	55 (84.6%)	79 (89.8%)	0.339
*Use of vitamin D, n (%)*	102	53 (81.5%)	49 (55.7%)	0.001
*Use of calcium, n (%)*	95	43 (66.2%)	52 (59.1%)	0.302
*Use of NSAIDS, n (%)*	71	39 (60.0%)	32 (36.4%)	0.004
*Use of antibiotics, n (%)*	128	57 (87.7%)	71 (80.7%)	0.246
*Use of corticosteroids, n (%)*	101	50 (76.9%)	51 (58.0%)	0.016
*Hemoglobin, g/dL*	12.87 ± 0.17	12.20 ± 0.28	13.39 ± 0.21	0.178
*Iron, ug/dL*	67.73 ± 4.10	73.22 ± 5.88	61.86 ± 5.62	0.242
*Ferritin, ng/mL*	102.05 ± 15.89	103.78 ± 21.65	99.65 ± 23.57	0.400
*Transferrin, mg/dL*	142.64 ± 13.77	107.17 ± 17.11	180.33 ± 20.65	0.026
*Albumin, g/dL*	4.17 ± 0.16	4.25 ± 0.37	4.12 ± 0.49	0.077
*Bilirubin, mg/dL*	1.67 ± 0.36	3.18 ± 0.80	0.53 ± 0.04	0.000
*Aspartate aminotransferase, U/L*	49.04 ± 5.74	76.82 ± 12.13	27.86 ± 2.29	0.000
*Alanine aminotransferase, U/L*	47.73 ± 5.00	72.32 ± 9.37	27.86 ± 2.29	0.000
*Alkaline phosphatase, U/L*	151.46 ± 13.97	221.38 ± 26.60	97.54 ± 10.74	0.000
*C-reactive protein, mg/dL*	3.06 ± 0.58	4.56 ± 1.23	68.24 ± 5.35	0.997
*Creatinine, mg/dL*	0.97 ± 0.04	1.04 ± 0.08	0.91 ± 0.04	0.006
*Parathyroid hormone, pg/mL*	53.10 ± 8.99	53.53 ± 6.08	52.70 ± 16.39	0.622
*Calcium, mg/dL*	9.30 ± 0.05	9.24 ± 0.09	9.35 ± 0.05	0.002
*Phosphorus, mg/dL*	3.34 ± 0.09	3.36 ± 0.15	3.31 ± 0.11	0.977
*Diagnosis of PSC, n (%)*	74	49 (75.4%)	25 (28.4%)	0.000

In multivariable analysis, PSC was an independent risk factor for vitamin D deficiency in the multivariate analysis (odds ratio [OR]: 7.56; 95% confidence interval [CI]: 2.39–24.08; *P* *=* 0.001) along with vitamin D supplementation (OR: 2.58; 95% CI: 1.57–9.19; *P* = 0.018) as shown in **Table 2****.**

**Table 2.** Stepwise logistic regression of risk factors for vitamin D deficiency*

Variables	Adjusted Odds Ratio	95% Confidence Interval	*P*
Use of vitamin D	2.58	1.57–9.19	0.018
Aspartate aminotransferase	1.01	0.95–1.05	0.913
PSC	7.56	2.39–24.08	0.001

*Variables included in the stepwise logistic regression were transferrin, bilirubin, aspartate aminotransferase, alanine aminotransferase, alkaline phosphatase, creatinine, calcium, use of vitamin D, use of non-steroidal anti-inflammatory drugs, use of corticosteroids and diagnosis of PSC.

## Discussion

In this study, we found that PSC is an independent risk factor for vitamin D deficiency in UC patients with IPAA. Abnormal vitamin D levels occurred frequently in patients with IPAA without PSC as the frequency of low levels of vitamin D and vitamin deficiency was 73.4% and 20.3%, respectively. These findings are in accordance with previous studies that reported rates of vitamin D deficiency in IPAA patients of 21.7% [17, 18]. Interestingly vitamin D levels were found to be much lower when patients had PSC, with low levels of vitamin D occurring in 91.9% of these patients, while vitamin D deficiency was found in 66.2% of patients.

There are limited data in the literature regarding vitamin D deficiency in patients with PSC. Jorgensen *et al*. evaluated levels of vitamin D among other fat-soluble vitamins in PSC patients [6). In that study, vitamin D levels were assessed in 143 patients with PSC without mention of UC, of which 56 PSC patients received ursodeoxycholic acid (UDCA) and did not have end-stage liver disease, while 87 patients had advanced PSC and were being evaluated for liver transplantation. The prevalence of vitamin D deficiency in the UDCA treatment group, which consisted of patients without end-stage liver disease, was 14%, while 57% of the patients in the pre-transplantation group had vitamin D deficiency [6]. However, vitamin D deficiency was considered levels less than 14–15 ng/dl, which is now known to be well below normal vitamin D levels.

It is well known that the skin is the main source of vitamin D through conversion of 7– dehydrocholesterol to vitamin D_3_ (cholecalciferol) by sunlight exposure. The vitamin D produced in the skin is transported to the liver bound to vitamin-D binding protein. In the liver, vitamin D undergoes the first hydroxylation to produce 25- hydroxy vitamin D_3_. Thus vitamin D metabolism is closely related to liver function. A second hydroxylation step then occurs in the kidney to form 1,25-hydroxyvitamin D_3_, which is the active vitamin D metabolite. Dietary intake of vitamin D_3_ is a smaller contribution to the total vitamin D pool, although this contribution may become important in geographic locations where the sunlight is less abundant, such as northern countries. Vitamin D acquired through the diet is absorbed mainly in the duodenum and jejunum and depends on bile-acids since it is a liposoluble vitamin [16, 19].

Cholestatic diseases such as primary biliary cirrhosis and PSC are associated with vitamin D deficiency. The main mechanism of vitamin D deficiency was proposed to be impaired intestinal absorption of vitamin D due to insufficient intraluminal bile acid concentrations [16, 20]. In the setting of significant cholestasis, even oral administration of larger doses of vitamin formulations may be inadequate, and periodic intramuscular administration of vitamins may be needed due to poor intestinal absorption [19–21].

Vitamin D deficiency has also been shown to be common in patients with chronic liver disease in general with a prevalence ranging 46% to 75% [11, 22–24]. There are several proposed mechanisms to explain vitamin D status in chronic liver disease including reduced exogenous exposure of patients to vitamin D sources (e.g. dietary, sunlight), intestinal malabsorption of dietary vitamin D, reduced endogenous production of vitamin D-binding protein and albumin in the liver (which are impaired in the presence of cirrhosis), impaired hepatic hydroxylation of vitamin D to 25(OH)D_3_ and increased catabolic removal of 25(OH)D_3_ [11].

Vitamin D deficiency can occur in early stages of PSC; however, the rates of vitamin D deficiency seem to be much higher when PSC reaches end-stage liver disease [6]. Although liver biopsy is the only way to definitely confirm cirrhosis and serum albumin is not indicated as a screening tool for cirrhosis, the evaluation of blood albumin levels can be used to help grade the severity of cirrhosis and further infer liver synthetic function [25]. In this study, the level of albumin in IPAA patients with PSC was found to be in the expected range for healthy individuals and was not statistically different from the IPAA patients without PSC (3.8 ± 0.1 *vs* 4.5 ± 0.3; *P* *=* 0.320), suggesting overall preserved liver synthetic function in the PSC patients studied. In addition, liver function tests in the PSC patients studied showed a cholestasis pattern, with aspartate aminotransferase of 75.7 ± 10.9, alananine aminotransferase of 75.9 ± 9.2, bilirubin of 2.4 ± 0.5 and alkaline phosphatase of 229.8 ± 25.3. This suggests that significant vitamin D deficiency in patients with IPAA and PSC occurs in the cholestatic phase of the disease before end-stage liver disease is established. This may be due to the fact that patients with UC and IPAA are already at risk for vitamin D deficiency due to adverse metabolic effects of the pouch construction, and dietary vitamin D sources may be significant for maintaining vitamin D levels. Cholestasis due to PSC may alter the thin balance that maintains vitamin D levels in these patients.

The association between the severity of the liver dysfunction and vitamin D deficiency was not assessed. Further prospective studies evaluating the association of vitamin D deficiency with the degree of liver dysfunction in PSC patients are needed to ascertain whether significant vitamin D deficiency already occurs in the cholestasis phase.

In this study, vitamin D supplementation remained a risk factor for vitamin D deficiency in the multivariate analysis (OR: 2.58; 95% CI: 1.57–9.19, *P* *=* 0.018). This can be explained by the fact that individuals with vitamin D deficiency are usually started on vitamin D supplementation. It may be interesting in the future to evaluate the exact dose and duration of vitamin D supplementation that could prevent vitamin D deficiency in these patients.

There are several limitations to this study. The sample size may have limited the power of the study. Since our center is a tertiary center, the high rates of vitamin D deficiency may reflect greater disease severity of the IBD population studied than the general IBD population. In addition, the geographic location of patients was not associated with vitamin D deficiency, but it needs to be taken into consideration that 134 of the 153 individuals included in this study (87.5%) were from northern locations.

Although there were no differences in vitamin D levels between races, conclusions about race are difficult to ascertain in this study given the way this information is entered into our database, with patients being divided as Hispanics and non-Hispanics only. Because this is a retrospective study, it was not possible to ascertain patient compliance to vitamin D supplementation. It will also be interesting to compare different doses of vitamin D supplementation in patients with IPAA and PSC in the future to establish the exact dose of vitamin D supplementation that would prevent vitamin D deficiency in these patients.

In conclusion, vitamin D deficiency was common in this patient population, suggesting the need for close evaluation of vitamin D levels. More studies are needed to clarify the actual mechanism by which PSC contributes to lower levels of vitamin D in IPAA patients.

*Conflict of interest statement:* none declared.
